# Association between hypoalbuminemia and complications after degenerative and deformity-correcting spinal surgeries: A systematic review and meta-analysis

**DOI:** 10.3389/fsurg.2022.1030539

**Published:** 2023-01-06

**Authors:** Xia Li, Haidong Li, Shufeng Huang, Yiping Pan

**Affiliations:** ^1^ Department of Orthopedics Nursing, First Affiliated Hospital of Huzhou University, Huzhou, China; ^2^ Department of Orthopedics, First Affiliated Hospital of Huzhou University, Huzhou, China

**Keywords:** albumin, nutrition, malnutrition, complications, surgery, spine

## Abstract

**Objective:**

The current review was designed to explore if hypoalbuminemia is associated with increased complications in patients undergoing spinal degenerative and deformities surgeries.

**Methods:**

The search for eligible studies was conducted on the databases of PubMed, Embase, Web of Science, and CENTRAL up to 20th June 2022. Complication rates were pooled to obtain odds ratio (OR) and 95% confidence intervals.

**Results:**

Thirteen studies were included. We found that hypoalbuminemia was significantly associated with increased risk of all complications (OR: 2.72 95% CI: 2.04, 3.63 *I*^2 ^= 58% *p* < 0.00001), mortality (OR: 7.73 95% CI: 3.81, 15.72 *I*^2 ^= 0% *p* < 0.00001), revision surgery (OR: 3.15 95% CI: 1.53, 6.48 *I*^2 ^= 87% *p* = 0.002), readmissions (OR: 1.96 95% CI: 1.29, 2.98 *I*^2 ^= 23% *p* = 0.02), surgical site infections (OR: 2.97 95% CI: 1.90, 4.63 *I*^2 ^= 38% *p* < 0.00001), wound complications (OR: 2.31 95% CI: 1.17, 4.56 *I*^2 ^= 48% *p* = 0.02), pulmonary complications (OR: 3.74 95% CI: 2.66, 5.26 *I*^2 ^= 0% *p* < 0.00001), renal complications (OR: 3.04 95% CI: 1.22, 7.54 *I*^2 ^= 0% *p* = 0.02), cardiac complications (OR: 4.33 95% CI: 2.14, 8.77 *I*^2 ^= 0% *p* < 0.0001), urinary tract infections (OR: 2.08 95% CI: 1.80, 2.41 *I*^2 ^= 0% *p* < 0.00001), and sepsis (OR: 4.95 95% CI: 1.87, 13.08 *I*^2 ^= 64% *p* = 0.01) as compared to those with normal albumin.

**Conclusion:**

Hypoalbuminemia is a significant risk factor for complications after spinal degenerative and deformity surgeries. Research is also needed on the role of nutritional support in improving outcomes after spinal degenerative and deformity surgeries.

**Systematic Review Registration:**

https://www.crd.york.ac.uk/prospero/, identifier: CRD42022340024.

## Introduction

The role of nutrition in the context of surgical interventions has gained much attention in the past decade (1–4). Malnutrition due to inadequate intake of calories, proteins, or other nutrients required for baseline tissue maintenance and repair is commonly recognized in surgical patients (5). Research suggests almost 60% of surgical patients are malnourished at the point of hospital admission which can have a significant impact on prognosis (6). The problem is further compounded by the fact that malnutrition is often under-recognized and under-treated by hospital staff possibly due to a lack of training and awareness (7). The presence of malnutrition has been recognized as an independent risk factor for complications in gastrointestinal, oncological, and orthopedic surgery patients wherein the adverse events can range from infections, wound complications, and renal complications to even heightened mortality (8). While several methods like serological markers, anthropometric measurements, and nutrition scoring tools are available to identify a malnourished patient, serum albumin level is one of the simplest and readily available markers which can rapidly indicate the nutritional status of an individual (9).

Spinal surgeries for degenerative diseases and deformities are being increasingly performed around the world. Improvements in technology and advances in surgical protocols have made fusion surgeries relatively safe over the years with high success rates. Nevertheless, the risk of complications is still in the range of 1%–20% (10). Any increase in complications adds to patient suffering and increases the length of hospital stay and medical expenditure. Therefore it is pertinent to identify risk factors for such complications so that timely and appropriate corrective measures can be taken to reduce their incidence.

Several studies in the literature have reported an association between low albumin and complication rates after spinal degenerative and deformities surgeries (11–13). However, evidence is conflicting with some studies showing a positive association between hypoalbuminemia and complications while others reporting no such relationship. Yoshida et al. (12) have demonstrated that hypoalbuminemia is a significant risk factor for deep infections, urinary tract infections, and revisions after lumbar spine surgeries. Elsamadicy et al. (13) also found similar association for adverse events and unplanned readmissions in patients undergoing surgery for spondylolisthesis. Contrastingly, Zhang et al. (14) in 2020 conducted a study on 602 patients and noted no association between hypoalbuminemia and surgical site infections (SSI) after spinal fusion surgeries. Similarly, Shoji et al. (15) have also noted no independent association between hypoalbuminemia and SSIs in spinal instrumentation surgery. Recently, Gretchen et al. (11) also could not find significant associations between hypoalbuminemia and adverse events, readmission rates, and revision surgeries in a cohort of 128 spinal deformity patients. Considering these variable results, there is a need for a quantitative analysis of published studies to generate high-quality evidence on the exact association between hypoalbuminemia and various complications after spinal degenerative and deformities surgeries. Such pooled data would provide a broad perspective to surgeons, enhancing their ability to identify patients at risk for complications and help prioritize management of the same. Therefore, the current review was designed to pool data from published studies and analyze the association between hypoalbuminemia and various complications in patients undergoing spinal degenerative and deformities surgeries.

## Material and methods

We initially registered the study on PROSPERO (CRD42022340024). Eligible studies for the review were sourced by formulating the inclusion criteria based on PECOS. We included studies conducted on patients undergoing any spinal degenerative or deformity correction surgeries (*Population*). The *Exposure* variable was the presence of perioperative hypoalbuminemia *Compared* with a control group of patients with normal albumin levels. The *Outcomes* were to be any complications. There was no restriction on *Study type* as long as they were comparative in design. To include maximum studies in the literature we did not define hypoalbuminemia ourselves and included all definitions used reported in the included studies.

Studies on trauma or oncological spinal surgery were excluded. Also, studies not dividing the population based on albumin were excluded. We also did not include studies with duplicate data, review articles, and case series.

The search for eligible studies was conducted on the databases of PubMed, Embase, Web of Science, and CENTRAL up to 20 June 2022 with the language of publication restricted to English. The PRISMA statement guidelines were followed for the review (16). The search strategy was based on the combination of the following keywords: “spinal surgery”; “lumbar surgery”; cervical spine surgery”; “spinal fusion”; “lumbar fusion”; “cervical fusion”; “albumin”; “hypoalbuminemia”; “malnutrition”; and “complications”. Further details specific to the PubMed search are available in Supplementary Table S1. The search queries were similar for other databases.

Once the search results were aggregated, they underwent software-based deduplication followed by the title and abstract screening of each article. Studies relevant to the review were downloaded for full-text analysis and studies fulfilling all criteria were included in the review. The entire process was carried out by two reviewers independent of each other. All disagreements were solved by consensus. The references of included studies were also cross-checked for additional articles.

Once selected, the studies underwent a standard data extraction process using a prepared form. We extracted details of study authors, year of publication, location and study database, type of surgery, sample size, demographic details, albumin cut-off used to define hypoalbuminemia, number of patients with hypoalbuminemia, complications assessed, follow-up duration, and outcome data. All reported complications were tabulated and then analyzed quantitatively provided at least three studies reported similar data.

The Newcastle Ottawa Scale (NOS) (17) was selected to judge the risk of bias amongst studies. The articles were judged on three domains: study population, comparability, and outcomes, and were given points based on preformatted questions. The total score of NOS ranges from 0 to 9.

### Statistical analysis

*“*Review Manager” [RevMan, version 5.3; Nordic Cochrane Centre (Cochrane Collaboration), Copenhagen, Denmark; 2014] was used for the meta-analysis. We extracted dichotomous data on complications and pooled them to obtain odds ratio (OR) with 95% CI. A random-effects model was used. We assessed inter-study heterogeneity using the *I*^2^ statistic. *I*^2 ^= 25%–50% meant low, 50%–75% meant medium, and more than 75% meant substantial heterogeneity. A funnel plot was used to detect publication bias. A leave-one-out analysis was performed to check for any change in the results on the exclusion of any study. Separate meta-analyses were performed for different complications. *P* values <0.05 were considered statistically significant.

## Results

**Figure 1:**
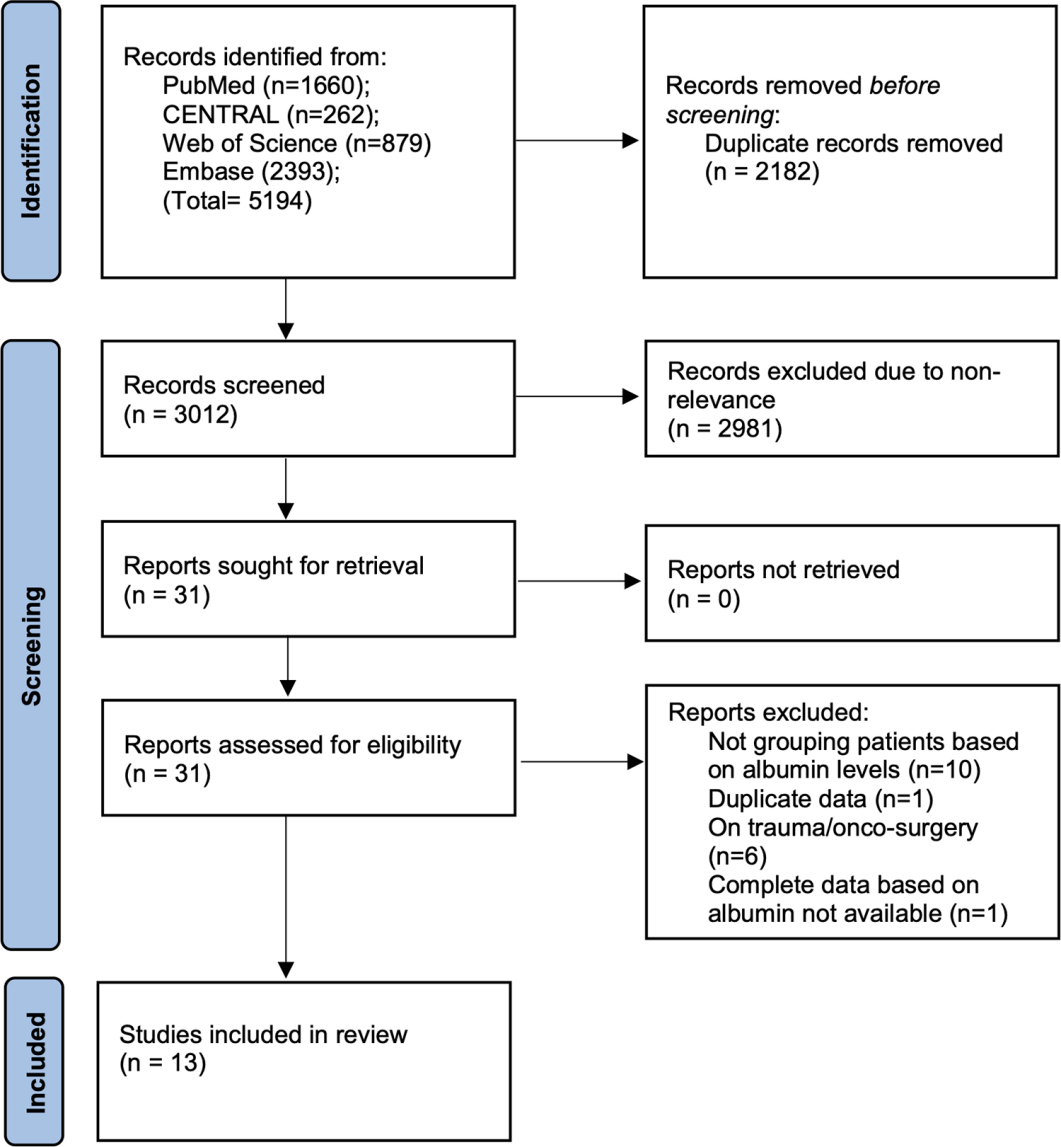
Study flow-chart.

There were 5,194 articles retrieved in the literature search (Figure 1). Out of those, 2,182 were duplicates and hence were removed. 3,012 articles underwent title and abstract screening and 31 studies were selected for full-text review. 18 studies were excluded with reasons and 13 were included in the review (10–15, 18–24).

The data extracted from the studies are presented in Table 1. The studies were published between 2014 and 2022. The majority of them were from the USA and used the American College of Surgeons National Surgical Quality Improvement Project database (ACS-NSQIP). The remaining studies were from Denmark, Japan, and China. There were small overlaps in study duration between the studies using the ACS-NSQIP database. The total sample size of all studies was 36,871 of which 2,267 (6.14%) had hypoalbuminemia. A cut-off of 3.5 g/dl of albumin was used to define hypoalbuminemia in all except two studies. One (18) used 3 g/dl as the criteria for hypoalbuminemia while another used age-stratified criteria with albumin cut-off ranging from 3.4 to 3.7 g/dl. To maintain homogeneity of the data, we excluded the study of Sebastian et al. (18) which used 3 g/dl from the meta-analysis and performed only a descriptive analysis. All except one study used preoperative albumin values. Almost all studies reported 30-day complication rates.

**Table 1:** Details of included studies.

Study	Location	Database	Surgery	Sample size	Age (years)	Male gender (%)	Albumin cut-off (g/dl)	Timing of measurement	Patients with low albumin	Complications assessed	Follow-up	NOS score
Gehrchen 2022 (11)	Denmark	Copenhagen University Hospital	Spinal deformity surgery	113	62	32	≥15 years: >3.7 ≥40 years: 3.6 ≥70 years: 3.4	Preoperative	20	Adverse events, readmission, revisions, mortality	30 days	8
Yoshida 2021 (12)	USA	PearlDiver (2007–2016)	Lumbar fusion or discectomy	20,229	NR	57.4	3.5	Preoperative	746	Deep infection, UTI, revision	NR	7
Elsamadicy 2021 (13)	USA	ACS-NSQIP (2010–2016)	Posterior lumbar decompression and fusion for spondylolisthesis	561	66	30.1	3.5	Preoperative	98	SSI, dehiscence, medical adverse events, reoperation, readmission	30 days	8
Zhang 2020 (14)	China	Zhujiang Hospital	Spinal fusion surgery	602	NR	41.5	3.5	Postoperative	438	SSI	30 days	8
He 2020 (10)	China	First Affiliated Hospital of Chongqing Medical University	Posterior lumbar fusion	544	56.9	43	3.5	Preoperative	13	Dehiscence, SSI	NR	7
Phan 2019 (21)	USA	ACS-NSQIP (2005–2012)	Elective posterior lumbar fusion	2,410	NR	43.6	3.5	Preoperative	159	Mortality, pulmonary, renal, CNS, cardiac complications, VTE, UTI, sepsis, wound complications	30 days	8
Ukogu 2018 (22)	USA	ACS-NSQIP (2011–2014)	Anterior lumbar interbody fusion	1,275	NR	44.8	3.5	Preoperative	53	Mortality, pulmonary, renal, cardiac complications, VTE, UTI, sepsis, wound complications, readmission, reoperation	30 days	8
Shoji 2018 (15)	Japan	Niigata University	Spinal fusion surgery	431	44.5	47	3.5	Preoperative	62	SSI	30 days	6
Phan 2018 (23)	USA	ACS-NSQIP (2010–2014)	Spinal deformity surgery	2,236	NR	42.1	3.5	Preoperative	192	Mortality, pulmonary, renal, cardiac complications, VTE, UTI, sepsis, wound complications, readmission, reoperation	30 days	8
Lee 2017 (24)	USA	ACS-NSQIP (2010–2014)	Posterior cervical fusion	1,573	NR	56.7	3.5	Preoperative	265	Mortality, pulmonary, renal, cardiac complications, VTE, UTI, sepsis, wound complications	30-days	8
Fu 2016 (19)	USA	ACS-NSQIP (2005–2010)	Anterior cervical discectomy and fusion	1,382	53.4	50.1	3.5	Preoperative	65	Mortality, pulmonary, renal, CNS, cardiac complications, VTE, UTI, sepsis, wound complications, reoperation	30-days	8
Sebastian 2015 (18)	USA	ACS-NSQIP (2005–2012)	Posterior cervical fusion/ decompression/ laminoplasty	5,441	59	58.6	3	Preoperative	142	SSI	30-days	8
Adogwa 2014 (20)	USA	Duke University Medical Center	Spinal fusion	74	57.1	48.6	3.5	Preoperative	14	SSI, sepsis, VTE, cardiac complications, stroke, UTI, pneumonia	NR	6

ACS-NSQIP, American College of Surgeons National Surgical Quality Improvement Project; CNS, Central nervous system; NOS, Newcastle Ottawa scale; SSI, Surgical site infection; UTI, urinary tract infection; VTE, venous thromboembolism.

### Meta-analysis

**Figure 2:**
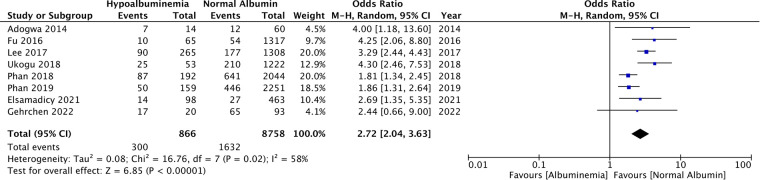
Meta-analysis of all complications between patients with hypoalbuminemia and normal albumin levels.

Eight studies reported the incidence of any complications in patients with hypoalbuminemia and normal albumin levels. On meta-analysis, it was noted that patients with hypoalbuminemia had significantly higher complication rates than those with normal albumin levels (OR: 2.72 95% CI: 2.04, 3.63 *I*^2 ^= 58% *p* < 0.00001) (Figure 2). There was no evidence of publication bias (Supplementary Figure S1).

**Figure 3:**
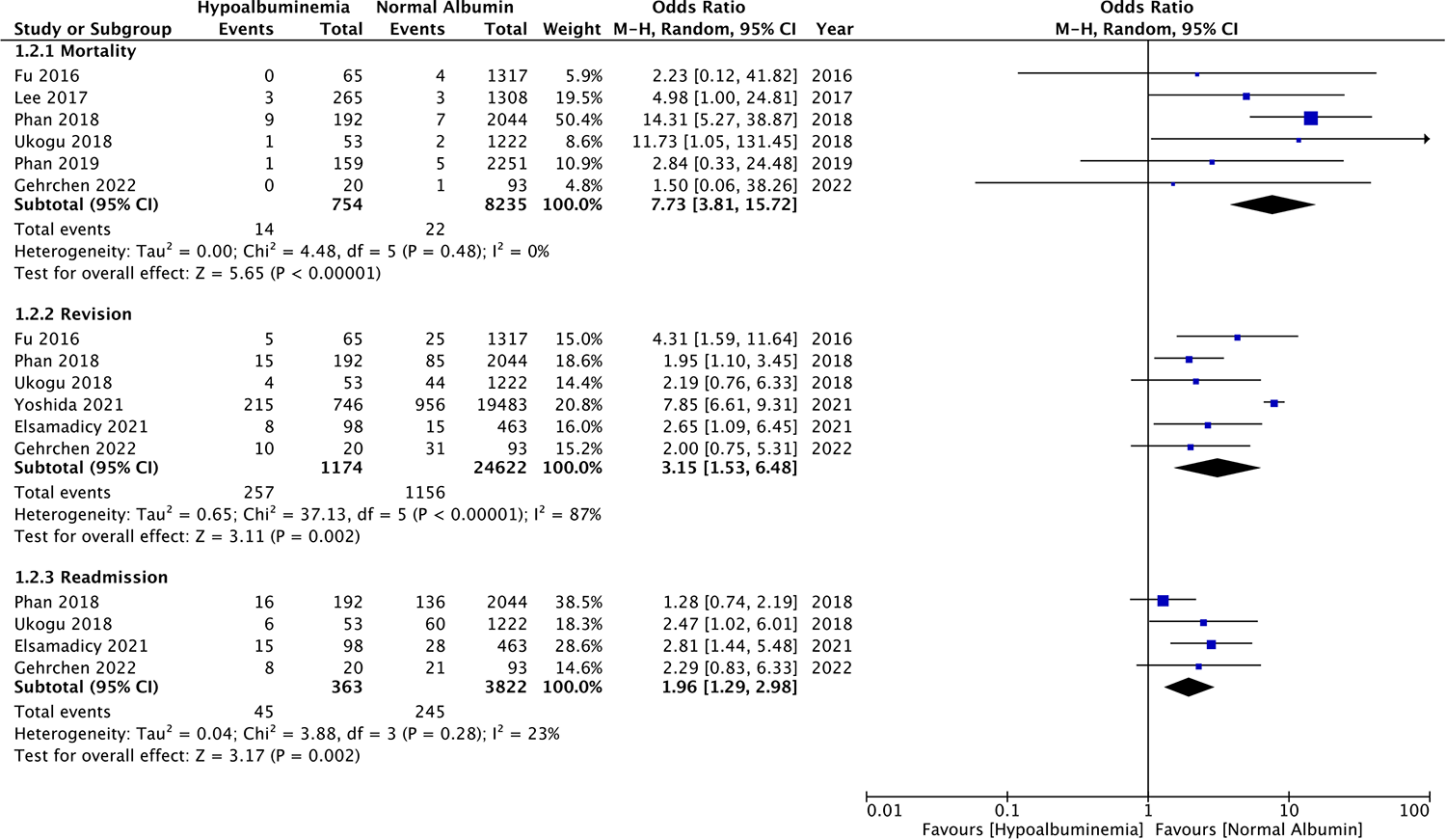
Meta-analysis of mortality, revision and readmissions between patients with hypoalbuminemia and normal albumin levels.

Data on 30-day mortality was reported by six studies. Pooled analysis showed significantly higher rates of mortality in patients with hypoalbuminemia as compared to those with normal albumin levels (OR: 7.73 95% CI: 3.81, 15.72 *I*^2 ^= 0% *p* < 0.00001) (Figure 3). Incidence of revision surgery and unplanned hospital readmissions was reported in six and four studies respectively. We found that hypoalbuminemia was significantly associated with increased odds of revision surgery (OR: 3.15 95% CI: 1.53, 6.48 *I*^2 ^= 87% *p* = 0.002) and unplanned hospital readmissions (OR: 1.96 95% CI: 1.29, 2.98 *I*^2 ^= 23% *p* = 0.02) (Figure 3).

**Figure 4:**
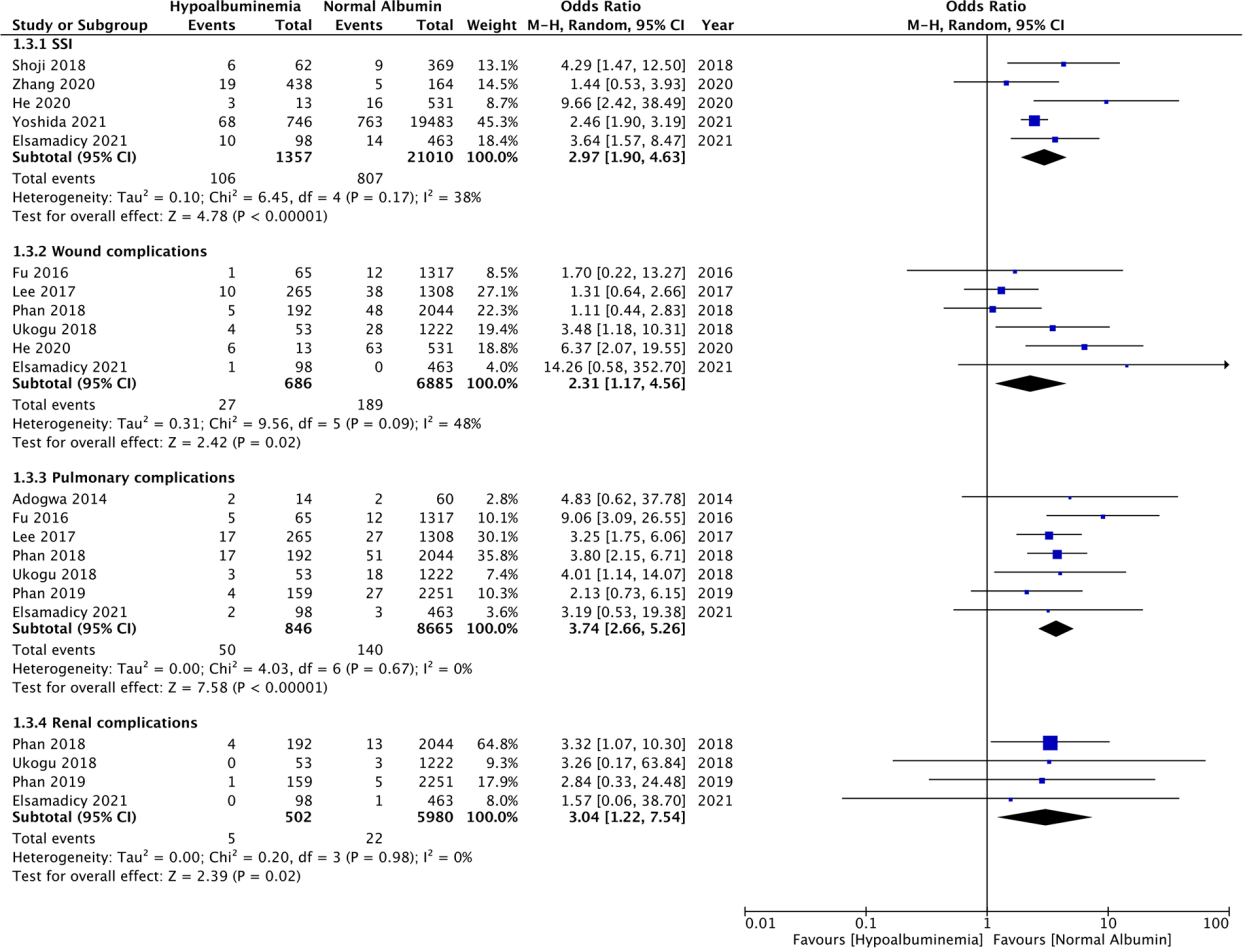
Meta-analysis of surgical site infections (SSI), wound complications, pulmonary complications and renal complications between patients with hypoalbuminemia and normal albumin levels.

The meta-analysis also showed that patients with hypoalbuminemia were at an increased risk of SSIs as compared to those with normal albumin levels (OR: 2.97 95% CI: 1.90, 4.63 *I*^2 ^= 38% *p* < 0.00001) (Figure 4). The study of Sebastian et al. (18) which was not included in the meta-analysis also reported significantly higher incidence of SSIs in patients with hypoalbuminemia as compared to those with normal albumin levels (hypoalbuminemia group: 10 SSIs/142 patients and normal albumin group: 150 SSIs/5,299 patients).

**Figure 5:**
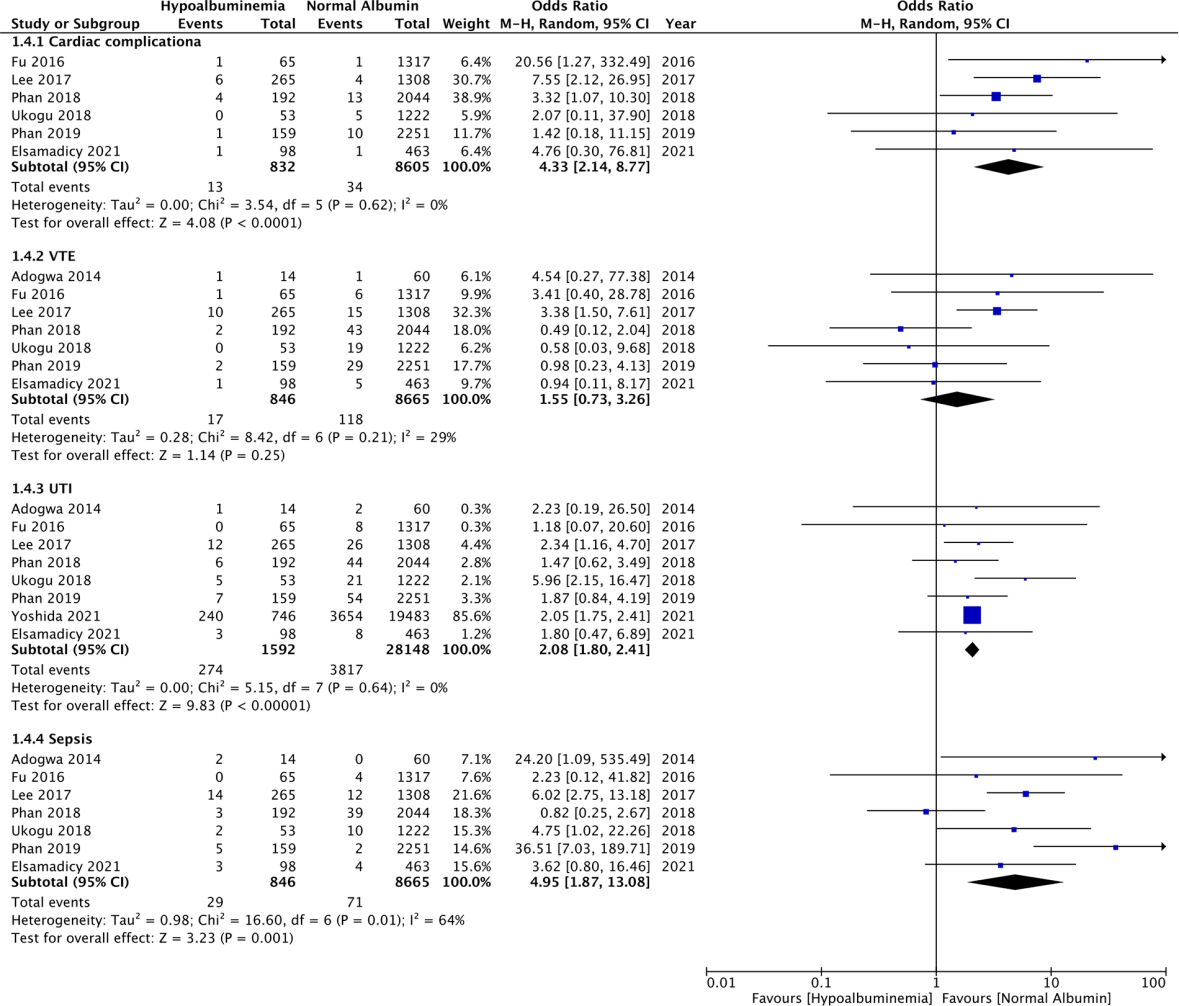
Meta-analysis of cardiac complications, venous thromboembolism (VTE), urinary tract infections (UTI) and sepsis between patients with hypoalbuminemia and normal albumin levels.

Similarly, wound complications (OR: 2.31 95% CI: 1.17, 4.56 *I*^2 ^= 48% *p* = 0.02), pulmonary complications (OR: 3.74 95% CI: 2.66, 5.26 *I*^2 ^= 0% *p* < 0.00001), and renal complications (OR: 3.04 95% CI: 1.22, 7.54 *I*^2 ^= 0% *p* = 0.02) were also significantly higher in patients with hypoalbuminemia (Figure 4). Meta-analysis also revealed that incidence of cardiac complications (OR: 4.33 95% CI: 2.14, 8.77 *I*^2 ^= 0% *p* < 0.0001), urinary tract infections (OR: 2.08 95% CI: 1.80, 2.41 *I*^2 ^= 0% *p* < 0.00001), and sepsis (OR: 4.95 95% CI: 1.87, 13.08 *I*^2 ^= 64% *p* = 0.01) was higher in patients with hypoalbuminemia as compared to controls (Figure 5). However, the incidence of venous thromboembolism (VTE) was not higher in patients with hypoalbuminemia (OR: 1.55 95% CI: 0.73, 3.26 *I*^2 ^= 29% *p* = 0.25) (Figure 5).

### Leave-one-out analysis

A few results changed during the leave-one-out analysis. The results of wound complications turned non-significant on exclusion of the studies of Ukogu et al. (22) (OR: 2.13 95% CI: 0.95, 4.78 *I*^2 ^= 52% *p* = 0.07) and He et al. (10) (OR: 1.69 95% CI: 0.97, 2.92 *I*^2 ^= 14% *p* = 0.06). Renal complications were not increased in patients with hypoalbuminemia on exclusion of Phan et al. (21) (OR: 2.57 95% CI: 0.56, 11.91 *I*^2 ^= 0% *p* = 0.23). Contrastingly on the exclusion of Phan et al. (21), there was a significantly increased risk of VTE noted in patients with hypoalbuminemia (OR: 2.28 95% CI: 1.24, 4.20 *I^2^*^ ^= 0% *p* = 0.008). All other results remain unchanged on the sequential exclusion of individual studies.

## Discussion

The current review of 13 studies with 36,871 patients is the first to assess the impact of hypoalbuminemia on complication rates after spinal degenerative and deformity correcting surgeries. We found that hypoalbuminemia is a significant risk factor for complication rates which increases the odds of early mortality, revision surgeries, unplanned readmissions, SSI, wound, pulmonary, renal, and cardiac complications along with UTIs and sepsis in patients undergoing spinal degenerative and deformity correcting surgeries.

Preoperative nutritional status as a marker of postoperative complications has received much attention in recent literature. In orthopedics, several studies have noted a positive association between malnutrition markers and the rate of postoperative complications (4, 25–27). Tsantes et al. (27) in a recent review of seven studies involving >250,000 patients found that malnutrition (defined by either albumin, transferrin, or total leucocyte count) was a significant predictor of prosthetic joint infections and SSI in patients undergoing total joint arthroplasty. Similarly, Yuwen et al. (25) in a meta-analysis of all orthopedic surgery patients noted a significantly increased risk of SSI with hypoalbuminemia. Wilson et al. (3) in a retrospective study of 377 hip fracture patients have shown that reduced albumin levels were associated with a significant increase in patient postoperative complications and early mortality. Similarly, Yagi et al. (2) in a study on 211 elderly patients undergoing surgical intervention for hip fractures found a statistically significant association between Controlling Nutritional Status (CONUT) scores (defined based on albumin, cholesterol, and total lymphocyte count) and risk of postoperative complications.

It is well accepted that different surgical procedures have different complication rates which can vary with the surgical site along with the nature and extent of the surgery. Focusing on spine surgeries, there is evidence that shows an association between malnutrition and complications after spinal surgeries as well. In a recently published study, Ushirozako et al. (26) retrospectively studied 115 patients undergoing different types of spinal surgeries and analyzed factors associated with SSI. The authors found that prognostic nutritional index (defined by albumin and total lymphocyte count) was a strong predictor of SSI in their cohort. The role of albumin as a singular marker has also received much attention. Gelfand et al. (1) in an analysis of 700 patients undergoing surgery for metastatic spinal disease have found a significant association between hypoalbuminemia and early mortality. Similar results have been obtained by Chen et al. (28) in a group of 128 patients with traumatic cervical spine injury. It must be noted that there are differences in patients undergoing spinal surgery for oncological or traumatic causes as opposed to elective surgeries for spinal deformities or degenerative diseases. Oncological patients are significantly immunocompromised secondary to cancer cachexia, chemotherapy, and radiation therapy which greatly elevates the risk of complications after any surgical procedure (1). Traumatic injuries are associated with a pro-inflammatory state resulting in excessive production of various inflammatory mediators and cytokines (29). In such cases, the inflammatory cytokines cause hepatic reprioritization of protein synthesis wherein the liver preferentially produces acute-phase reactants as opposed to albumin. Therefore, in these individuals using hypoalbuminemia as a surrogate for malnutrition may not be correct (9).

Consequently, our review excluded studies on oncological or trauma-related spinal surgeries to obtain a more homogenous group of elective spinal surgery studies which could be analyzed together. The results of multiple analyses showed that hypoalbuminemia was indeed a significant risk factor for complications after spinal degenerative and deformity correcting surgeries. There was a two to four-fold increased risk of all complications, revision surgeries, unplanned readmissions, SSI, wound, pulmonary, renal, cardiac complications, UTIs, and sepsis in individuals with hypoalbuminemia. Furthermore, the meta-analysis showed a 7.7 times increased risk of early mortality in patients with hypoalbuminemia. The direction of the results was more or less congruent across studies with some variations in the strength of the association. The strength of our analysis lies in the stability of most results on leave-one-out analysis and lack of publication bias. Few results like that of wound and renal complications turned non-significant on the exclusion of singular studies. Nevertheless, the direction of the association was positive indicating a higher risk of such complications with hypoalbuminemia. Of note, is that a few studies like Gehrchen et al. (11) did not find statistically significant associations between hypoalbuminemia and complication rates. This can be noted in the forest plot of our analysis. Nevertheless, the direction of the results of this study too was indicative of increased complication rates with hypoalbuminemia and it is plausible that the small sample size of the study may have prevented significant associations.

Baseline differences between patients with normal and abnormal albumin levels can be an important confounder in the incidence of complications. Gehrchen et al. (11) in their study found that hypoalbuminemia patients had more comorbidities and significantly lower hemoglobin levels than those with normal albumin levels, both of which could increase the risk of complications. Similarly, Elsamadicy et al. (13) noted that their malnourished cohort was older and more likely to have impaired functional status, diabetes mellitus, lung disease, hypertension, anemia, and bleeding disorder as compared to those with normal albumin levels. All of these factors could contribute to the risk of complications after surgery. In our review, only one study (13) used propensity-score matching to adjust for baseline differences between the study groups. The current results should therefore be interpreted with caution considering the baseline difference amongst the study groups which could have confounded the risk of complications.

The clinical implications of our findings are that identification and correction of pre-operative albumin levels in patients undergoing spinal degenerative and deformity surgeries could reduce the risk of complications. Results from hip fracture populations have shown that preoperative nutritional supplements can reduce the risk of adverse events after surgery (30). However, data on elective spinal surgeries are scarce. A recent study by Zhang et al. (14) showed that postoperative administration of albumin increases the risk of SSI and cautioned clinicians against blind administration of albumin in all cases of postoperatively measured hypoalbuminemia. On the other hand, Oe et al. (31) in a small retrospective study have shown that nutritional support could reduce postoperative medical complications in adult spinal deformity patients. Nevertheless, current research is limited, and based on our results further studies are needed to explore the role of malnutrition correction in reducing complications after spinal degenerative and deformity surgeries.

Our review has some limitations. Firstly, most data were derived from a single dataset derived from the USA. This limits the generalizability of current evidence. There was also a small overlap (2 years) in the study period in some of the included studies and it is plausible that some patients may be counted twice. Secondly, the analysis in most studies was restricted to 30-day complication rates and the impact of hypoalbuminemia on long-term complications remains unknown. Thirdly, as mentioned earlier, all complications can be confounded by several baseline variables which were not accounted for in our analysis. Fourthly, despite including 13 studies in the review, the number of studies in the meta-analysis was low as all complication rates were not universally reported. This reduced the statistical power of individual analyses. Fifthly, albumin levels are related to presence of liver and kidney diseases. Due to unavailability of data, it was not possible for this review to segregate outcomes between patients with and without liver or kidney dysfunction. Lastly, while we aimed to include a homogenous group of studies, there were still some inter-study variations in terms of the site and extent of surgery which could have resulted in moderate heterogeneity in some of our analyses. Also, by including the entire ambit of degenerative and deformity surgeries, the review itself introduced heterogeneity in the review as the operation time, blood loss, extent of surgery, and complication rates can vary between the two set of procedures. This is one of the important limitation while interpreting the results. Additionally, one study (14) measured albumin postoperatively while all others measured it preoperatively. The impact of this study on the results was assessed using a sensitivity analysis which did not demonstrate any change in the results of any analysis.

## Conclusions

Hypoalbuminemia is a significant risk factor for complications after spinal degenerative and deformity surgeries. Patients with hypoalbuminemia are at an increased risk of all complications, revision surgeries, unplanned readmissions, SSI, wound, pulmonary, renal, cardiac complications, UTIs, and sepsis. Current evidence could be skewed due to baseline confounding. Future studies should take into account baseline variables while assessing the risk of complications with hypoalbuminemia. Research is also needed on the role of nutritional support in improving outcomes after spinal degenerative and deformity surgeries.

## Data Availability

The original contributions presented in the study are included in the article/**Supplementary Material**, further inquiries can be directed to the corresponding author/s.
