# Supplementary material for: Association between hypoalbuminemia and complications after degenerative and deformity-correcting spinal surgeries: A systematic review and meta-analysis
Source: Front Surg. 2023 Jan 6;9:1030539. doi: 10.3389/fsurg.2022.1030539 (PMC9852605; doi:10.3389/fsurg.2022.1030539)
Supplement: Supplementary file 1 [file Table1.docx]

Supplementary Table 1: Search strategy

| **Query** | **Search Details** |
| --- | --- |
| ((((spinal surgery) OR (lumbar surgery)) OR (cervical spine surgery)) AND (albumin)) AND (complications) | ((("spinal"[All Fields] OR "spinalization"[All Fields] OR "spinalized"[All Fields] OR "spinally"[All Fields] OR "spinals"[All Fields]) AND ("surgery"[MeSH Subheading] OR "surgery"[All Fields] OR "surgical procedures, operative"[MeSH Terms] OR ("surgical"[All Fields] AND "procedures"[All Fields] AND "operative"[All Fields]) OR "operative surgical procedures"[All Fields] OR "general surgery"[MeSH Terms] OR ("general"[All Fields] AND "surgery"[All Fields]) OR "general surgery"[All Fields] OR "surgery s"[All Fields] OR "surgerys"[All Fields] OR "surgeries"[All Fields])) OR (("lumbarised"[All Fields] OR "lumbarization"[All Fields] OR "lumbarized"[All Fields] OR "lumbars"[All Fields] OR "lumbosacral region"[MeSH Terms] OR ("lumbosacral"[All Fields] AND "region"[All Fields]) OR "lumbosacral region"[All Fields] OR "lumbar"[All Fields]) AND ("surgery"[MeSH Subheading] OR "surgery"[All Fields] OR "surgical procedures, operative"[MeSH Terms] OR ("surgical"[All Fields] AND "procedures"[All Fields] AND "operative"[All Fields]) OR "operative surgical procedures"[All Fields] OR "general surgery"[MeSH Terms] OR ("general"[All Fields] AND "surgery"[All Fields]) OR "general surgery"[All Fields] OR "surgery s"[All Fields] OR "surgerys"[All Fields] OR "surgeries"[All Fields])) OR (("cervical vertebrae"[MeSH Terms] OR ("cervical"[All Fields] AND "vertebrae"[All Fields]) OR "cervical vertebrae"[All Fields] OR ("cervical"[All Fields] AND "spine"[All Fields]) OR "cervical spine"[All Fields]) AND ("surgery"[MeSH Subheading] OR "surgery"[All Fields] OR "surgical procedures, operative"[MeSH Terms] OR ("surgical"[All Fields] AND "procedures"[All Fields] AND "operative"[All Fields]) OR "operative surgical procedures"[All Fields] OR "general surgery"[MeSH Terms] OR ("general"[All Fields] AND "surgery"[All Fields]) OR "general surgery"[All Fields] OR "surgery s"[All Fields] OR "surgerys"[All Fields] OR "surgeries"[All Fields]))) AND ("albumin s"[All Fields] OR "albumine"[All Fields] OR "albumines"[All Fields] OR "albumins"[MeSH Terms] OR "albumins"[All Fields] OR "albumin"[All Fields]) AND ("complicances"[All Fields] OR "complicate"[All Fields] OR "complicated"[All Fields] OR "complicates"[All Fields] OR "complicating"[All Fields] OR "complication"[All Fields] OR "complication s"[All Fields] OR "complications"[MeSH Subheading] OR "complications"[All Fields]) |
| ((((spinal fusion) OR (lumbar fusion)) OR (cervical fusion)) AND (albumin)) AND (complications) | ("spinal fusion"[MeSH Terms] OR ("spinal"[All Fields] AND "fusion"[All Fields]) OR "spinal fusion"[All Fields] OR (("lumbarised"[All Fields] OR "lumbarization"[All Fields] OR "lumbarized"[All Fields] OR "lumbars"[All Fields] OR "lumbosacral region"[MeSH Terms] OR ("lumbosacral"[All Fields] AND "region"[All Fields]) OR "lumbosacral region"[All Fields] OR "lumbar"[All Fields]) AND ("fusions"[All Fields] OR "gene fusion"[MeSH Terms] OR ("gene"[All Fields] AND "fusion"[All Fields]) OR "gene fusion"[All Fields] OR "fusion"[All Fields])) OR (("cervic"[All Fields] OR "cervicals"[All Fields] OR "cervices"[All Fields] OR "neck"[MeSH Terms] OR "neck"[All Fields] OR "cervical"[All Fields] OR "uterine cervicitis"[MeSH Terms] OR ("uterine"[All Fields] AND "cervicitis"[All Fields]) OR "uterine cervicitis"[All Fields] OR "cervicitis"[All Fields]) AND ("fusions"[All Fields] OR "gene fusion"[MeSH Terms] OR ("gene"[All Fields] AND "fusion"[All Fields]) OR "gene fusion"[All Fields] OR "fusion"[All Fields]))) AND ("albumin s"[All Fields] OR "albumine"[All Fields] OR "albumines"[All Fields] OR "albumins"[MeSH Terms] OR "albumins"[All Fields] OR "albumin"[All Fields]) AND ("complicances"[All Fields] OR "complicate"[All Fields] OR "complicated"[All Fields] OR "complicates"[All Fields] OR "complicating"[All Fields] OR "complication"[All Fields] OR "complication s"[All Fields] OR "complications"[MeSH Subheading] OR "complications"[All Fields]) |
| ((((spinal surgery) OR (lumbar surgery)) OR (cervical spine surgery)) AND (malnutrition)) AND (complications) | ((("spinal"[All Fields] OR "spinalization"[All Fields] OR "spinalized"[All Fields] OR "spinally"[All Fields] OR "spinals"[All Fields]) AND ("surgery"[MeSH Subheading] OR "surgery"[All Fields] OR "surgical procedures, operative"[MeSH Terms] OR ("surgical"[All Fields] AND "procedures"[All Fields] AND "operative"[All Fields]) OR "operative surgical procedures"[All Fields] OR "general surgery"[MeSH Terms] OR ("general"[All Fields] AND "surgery"[All Fields]) OR "general surgery"[All Fields] OR "surgery s"[All Fields] OR "surgerys"[All Fields] OR "surgeries"[All Fields])) OR (("lumbarised"[All Fields] OR "lumbarization"[All Fields] OR "lumbarized"[All Fields] OR "lumbars"[All Fields] OR "lumbosacral region"[MeSH Terms] OR ("lumbosacral"[All Fields] AND "region"[All Fields]) OR "lumbosacral region"[All Fields] OR "lumbar"[All Fields]) AND ("surgery"[MeSH Subheading] OR "surgery"[All Fields] OR "surgical procedures, operative"[MeSH Terms] OR ("surgical"[All Fields] AND "procedures"[All Fields] AND "operative"[All Fields]) OR "operative surgical procedures"[All Fields] OR "general surgery"[MeSH Terms] OR ("general"[All Fields] AND "surgery"[All Fields]) OR "general surgery"[All Fields] OR "surgery s"[All Fields] OR "surgerys"[All Fields] OR "surgeries"[All Fields])) OR (("cervical vertebrae"[MeSH Terms] OR ("cervical"[All Fields] AND "vertebrae"[All Fields]) OR "cervical vertebrae"[All Fields] OR ("cervical"[All Fields] AND "spine"[All Fields]) OR "cervical spine"[All Fields]) AND ("surgery"[MeSH Subheading] OR "surgery"[All Fields] OR "surgical procedures, operative"[MeSH Terms] OR ("surgical"[All Fields] AND "procedures"[All Fields] AND "operative"[All Fields]) OR "operative surgical procedures"[All Fields] OR "general surgery"[MeSH Terms] OR ("general"[All Fields] AND "surgery"[All Fields]) OR "general surgery"[All Fields] OR "surgery s"[All Fields] OR "surgerys"[All Fields] OR "surgeries"[All Fields]))) AND ("malnutrition"[MeSH Terms] OR "malnutrition"[All Fields] OR "malnutrition s"[All Fields] OR "malnutritional"[All Fields] OR "malnutritions"[All Fields]) AND ("complicances"[All Fields] OR "complicate"[All Fields] OR "complicated"[All Fields] OR "complicates"[All Fields] OR "complicating"[All Fields] OR "complication"[All Fields] OR "complication s"[All Fields] OR "complications"[MeSH Subheading] OR "complications"[All Fields]) |
| ((((spinal fusion) OR (lumbar fusion)) OR (cervical fusion)) AND (malnutrition)) AND (complications) | ("spinal fusion"[MeSH Terms] OR ("spinal"[All Fields] AND "fusion"[All Fields]) OR "spinal fusion"[All Fields] OR (("lumbarised"[All Fields] OR "lumbarization"[All Fields] OR "lumbarized"[All Fields] OR "lumbars"[All Fields] OR "lumbosacral region"[MeSH Terms] OR ("lumbosacral"[All Fields] AND "region"[All Fields]) OR "lumbosacral region"[All Fields] OR "lumbar"[All Fields]) AND ("fusions"[All Fields] OR "gene fusion"[MeSH Terms] OR ("gene"[All Fields] AND "fusion"[All Fields]) OR "gene fusion"[All Fields] OR "fusion"[All Fields])) OR (("cervic"[All Fields] OR "cervicals"[All Fields] OR "cervices"[All Fields] OR "neck"[MeSH Terms] OR "neck"[All Fields] OR "cervical"[All Fields] OR "uterine cervicitis"[MeSH Terms] OR ("uterine"[All Fields] AND "cervicitis"[All Fields]) OR "uterine cervicitis"[All Fields] OR "cervicitis"[All Fields]) AND ("fusions"[All Fields] OR "gene fusion"[MeSH Terms] OR ("gene"[All Fields] AND "fusion"[All Fields]) OR "gene fusion"[All Fields] OR "fusion"[All Fields]))) AND ("malnutrition"[MeSH Terms] OR "malnutrition"[All Fields] OR "malnutrition s"[All Fields] OR "malnutritional"[All Fields] OR "malnutritions"[All Fields]) AND ("complicances"[All Fields] OR "complicate"[All Fields] OR "complicated"[All Fields] OR "complicates"[All Fields] OR "complicating"[All Fields] OR "complication"[All Fields] OR "complication s"[All Fields] OR "complications"[MeSH Subheading] OR "complications"[All Fields]) |
| ((((spinal surgery) OR (lumbar surgery)) OR (cervical spine surgery)) AND (hypoalbuminemia)) AND (complications) | ((("spinal"[All Fields] OR "spinalization"[All Fields] OR "spinalized"[All Fields] OR "spinally"[All Fields] OR "spinals"[All Fields]) AND ("surgery"[MeSH Subheading] OR "surgery"[All Fields] OR "surgical procedures, operative"[MeSH Terms] OR ("surgical"[All Fields] AND "procedures"[All Fields] AND "operative"[All Fields]) OR "operative surgical procedures"[All Fields] OR "general surgery"[MeSH Terms] OR ("general"[All Fields] AND "surgery"[All Fields]) OR "general surgery"[All Fields] OR "surgery s"[All Fields] OR "surgerys"[All Fields] OR "surgeries"[All Fields])) OR (("lumbarised"[All Fields] OR "lumbarization"[All Fields] OR "lumbarized"[All Fields] OR "lumbars"[All Fields] OR "lumbosacral region"[MeSH Terms] OR ("lumbosacral"[All Fields] AND "region"[All Fields]) OR "lumbosacral region"[All Fields] OR "lumbar"[All Fields]) AND ("surgery"[MeSH Subheading] OR "surgery"[All Fields] OR "surgical procedures, operative"[MeSH Terms] OR ("surgical"[All Fields] AND "procedures"[All Fields] AND "operative"[All Fields]) OR "operative surgical procedures"[All Fields] OR "general surgery"[MeSH Terms] OR ("general"[All Fields] AND "surgery"[All Fields]) OR "general surgery"[All Fields] OR "surgery s"[All Fields] OR "surgerys"[All Fields] OR "surgeries"[All Fields])) OR (("cervical vertebrae"[MeSH Terms] OR ("cervical"[All Fields] AND "vertebrae"[All Fields]) OR "cervical vertebrae"[All Fields] OR ("cervical"[All Fields] AND "spine"[All Fields]) OR "cervical spine"[All Fields]) AND ("surgery"[MeSH Subheading] OR "surgery"[All Fields] OR "surgical procedures, operative"[MeSH Terms] OR ("surgical"[All Fields] AND "procedures"[All Fields] AND "operative"[All Fields]) OR "operative surgical procedures"[All Fields] OR "general surgery"[MeSH Terms] OR ("general"[All Fields] AND "surgery"[All Fields]) OR "general surgery"[All Fields] OR "surgery s"[All Fields] OR "surgerys"[All Fields] OR "surgeries"[All Fields]))) AND ("hypoalbuminaemia"[All Fields] OR "hypoalbuminemia"[MeSH Terms] OR "hypoalbuminemia"[All Fields]) AND ("complicances"[All Fields] OR "complicate"[All Fields] OR "complicated"[All Fields] OR "complicates"[All Fields] OR "complicating"[All Fields] OR "complication"[All Fields] OR "complication s"[All Fields] OR "complications"[MeSH Subheading] OR "complications"[All Fields]) |
| ((((spinal fusion) OR (lumbar fusion)) OR (cervical fusion)) AND (hypoalbuminemia)) AND (complications) | ("spinal fusion"[MeSH Terms] OR ("spinal"[All Fields] AND "fusion"[All Fields]) OR "spinal fusion"[All Fields] OR (("lumbarised"[All Fields] OR "lumbarization"[All Fields] OR "lumbarized"[All Fields] OR "lumbars"[All Fields] OR "lumbosacral region"[MeSH Terms] OR ("lumbosacral"[All Fields] AND "region"[All Fields]) OR "lumbosacral region"[All Fields] OR "lumbar"[All Fields]) AND ("fusions"[All Fields] OR "gene fusion"[MeSH Terms] OR ("gene"[All Fields] AND "fusion"[All Fields]) OR "gene fusion"[All Fields] OR "fusion"[All Fields])) OR (("cervic"[All Fields] OR "cervicals"[All Fields] OR "cervices"[All Fields] OR "neck"[MeSH Terms] OR "neck"[All Fields] OR "cervical"[All Fields] OR "uterine cervicitis"[MeSH Terms] OR ("uterine"[All Fields] AND "cervicitis"[All Fields]) OR "uterine cervicitis"[All Fields] OR "cervicitis"[All Fields]) AND ("fusions"[All Fields] OR "gene fusion"[MeSH Terms] OR ("gene"[All Fields] AND "fusion"[All Fields]) OR "gene fusion"[All Fields] OR "fusion"[All Fields]))) AND ("hypoalbuminaemia"[All Fields] OR "hypoalbuminemia"[MeSH Terms] OR "hypoalbuminemia"[All Fields]) AND ("complicances"[All Fields] OR "complicate"[All Fields] OR "complicated"[All Fields] OR "complicates"[All Fields] OR "complicating"[All Fields] OR "complication"[All Fields] OR "complication s"[All Fields] OR "complications"[MeSH Subheading] OR "complications"[All Fields]) |
| ((((spinal surgery) OR (lumbar surgery)) OR (cervical spine surgery)) AND (nutrition)) AND (complications) | ((("spinal"[All Fields] OR "spinalization"[All Fields] OR "spinalized"[All Fields] OR "spinally"[All Fields] OR "spinals"[All Fields]) AND ("surgery"[MeSH Subheading] OR "surgery"[All Fields] OR "surgical procedures, operative"[MeSH Terms] OR ("surgical"[All Fields] AND "procedures"[All Fields] AND "operative"[All Fields]) OR "operative surgical procedures"[All Fields] OR "general surgery"[MeSH Terms] OR ("general"[All Fields] AND "surgery"[All Fields]) OR "general surgery"[All Fields] OR "surgery s"[All Fields] OR "surgerys"[All Fields] OR "surgeries"[All Fields])) OR (("lumbarised"[All Fields] OR "lumbarization"[All Fields] OR "lumbarized"[All Fields] OR "lumbars"[All Fields] OR "lumbosacral region"[MeSH Terms] OR ("lumbosacral"[All Fields] AND "region"[All Fields]) OR "lumbosacral region"[All Fields] OR "lumbar"[All Fields]) AND ("surgery"[MeSH Subheading] OR "surgery"[All Fields] OR "surgical procedures, operative"[MeSH Terms] OR ("surgical"[All Fields] AND "procedures"[All Fields] AND "operative"[All Fields]) OR "operative surgical procedures"[All Fields] OR "general surgery"[MeSH Terms] OR ("general"[All Fields] AND "surgery"[All Fields]) OR "general surgery"[All Fields] OR "surgery s"[All Fields] OR "surgerys"[All Fields] OR "surgeries"[All Fields])) OR (("cervical vertebrae"[MeSH Terms] OR ("cervical"[All Fields] AND "vertebrae"[All Fields]) OR "cervical vertebrae"[All Fields] OR ("cervical"[All Fields] AND "spine"[All Fields]) OR "cervical spine"[All Fields]) AND ("surgery"[MeSH Subheading] OR "surgery"[All Fields] OR "surgical procedures, operative"[MeSH Terms] OR ("surgical"[All Fields] AND "procedures"[All Fields] AND "operative"[All Fields]) OR "operative surgical procedures"[All Fields] OR "general surgery"[MeSH Terms] OR ("general"[All Fields] AND "surgery"[All Fields]) OR "general surgery"[All Fields] OR "surgery s"[All Fields] OR "surgerys"[All Fields] OR "surgeries"[All Fields]))) AND ("nutrition s"[All Fields] OR "nutritional status"[MeSH Terms] OR ("nutritional"[All Fields] AND "status"[All Fields]) OR "nutritional status"[All Fields] OR "nutrition"[All Fields] OR "nutritional sciences"[MeSH Terms] OR ("nutritional"[All Fields] AND "sciences"[All Fields]) OR "nutritional sciences"[All Fields] OR "nutritional"[All Fields] OR "nutritionals"[All Fields] OR "nutritions"[All Fields] OR "nutritive"[All Fields]) AND ("complicances"[All Fields] OR "complicate"[All Fields] OR "complicated"[All Fields] OR "complicates"[All Fields] OR "complicating"[All Fields] OR "complication"[All Fields] OR "complication s"[All Fields] OR "complications"[MeSH Subheading] OR "complications"[All Fields]) |
| ((((spinal fusion) OR (lumbar fusion)) OR (cervical fusion)) AND (nutrition)) AND (complications) | ("spinal fusion"[MeSH Terms] OR ("spinal"[All Fields] AND "fusion"[All Fields]) OR "spinal fusion"[All Fields] OR (("lumbarised"[All Fields] OR "lumbarization"[All Fields] OR "lumbarized"[All Fields] OR "lumbars"[All Fields] OR "lumbosacral region"[MeSH Terms] OR ("lumbosacral"[All Fields] AND "region"[All Fields]) OR "lumbosacral region"[All Fields] OR "lumbar"[All Fields]) AND ("fusions"[All Fields] OR "gene fusion"[MeSH Terms] OR ("gene"[All Fields] AND "fusion"[All Fields]) OR "gene fusion"[All Fields] OR "fusion"[All Fields])) OR (("cervic"[All Fields] OR "cervicals"[All Fields] OR "cervices"[All Fields] OR "neck"[MeSH Terms] OR "neck"[All Fields] OR "cervical"[All Fields] OR "uterine cervicitis"[MeSH Terms] OR ("uterine"[All Fields] AND "cervicitis"[All Fields]) OR "uterine cervicitis"[All Fields] OR "cervicitis"[All Fields]) AND ("fusions"[All Fields] OR "gene fusion"[MeSH Terms] OR ("gene"[All Fields] AND "fusion"[All Fields]) OR "gene fusion"[All Fields] OR "fusion"[All Fields]))) AND ("nutrition s"[All Fields] OR "nutritional status"[MeSH Terms] OR ("nutritional"[All Fields] AND "status"[All Fields]) OR "nutritional status"[All Fields] OR "nutrition"[All Fields] OR "nutritional sciences"[MeSH Terms] OR ("nutritional"[All Fields] AND "sciences"[All Fields]) OR "nutritional sciences"[All Fields] OR "nutritional"[All Fields] OR "nutritionals"[All Fields] OR "nutritions"[All Fields] OR "nutritive"[All Fields]) AND ("complicances"[All Fields] OR "complicate"[All Fields] OR "complicated"[All Fields] OR "complicates"[All Fields] OR "complicating"[All Fields] OR "complication"[All Fields] OR "complication s"[All Fields] OR "complications"[MeSH Subheading] OR "complications"[All Fields]) |
